# Supplementary material for: Advancing equitable access to digital mental health in the Asia-Pacific region in the context of the COVID-19 pandemic and beyond: A modified Delphi consensus study
Source: PLOS Glob Public Health. 2024 Jun 10;4(6):e0002661. doi: 10.1371/journal.pgph.0002661 (PMC11164385; doi:10.1371/journal.pgph.0002661)
Supplement: S1 Checklist — (DOCX) [file pgph.0002661.s001.docx]

Inclusivity in global research

PLOS’ policy on inclusivity in global research aims to improve transparency in the reporting of research performed outside of researchers’ own country or community and ensures that PLOS publications reporting global research adhere to high standards for research ethics and authorship. Authors of relevant research articles may be asked to complete the questionnaire below, which outlines ethical, cultural, and scientific considerations specific to inclusivity in global research. This questionnaire may be requested when researchers have travelled to a different country to conduct research, if research uses samples collected in another country, research with Indigenous populations or their lands, or if research is on cultural artefacts. Researchers travelling to another country solely to use laboratory equipment will not normally be required to complete the questionnaire. However, the questionnaire can be requested at the journal’s discretion for any submission – if you have been requested to complete this questionnaire by the PLOS journal you submitted to, please do so.

Please complete the questionnaire below and include this as a Supporting Information file with your manuscript. Note that if your paper is accepted for publication, this checklist will be published with your article in the supporting information files. Please ensure that you reference the checklist in the main body of your manuscript. We suggest adding a subsection ‘Inclusivity in global research’ to your Methods section and adding the following sentence: “Additional information regarding the ethical, cultural, and scientific considerations specific to inclusivity in global research is included in the Supporting Information (SX Checklist)”

The questions have been designed to be applicable to a wide range of study types, and there are subsections for both human subjects research and non-human subjects research. If any of the questions are not relevant to your research please mark them as “N/A” as appropriate.

**Ethical considerations, permits and authorship**

*This section is applicable to all research types.*

Provide details as to who granted permissions and/or consent for the study to take place in the Methods section of your manuscript. This should include the names of **all** ethics boards, governmental organizations, community leaders or other bodies that provided approval for the study. If individuals provided approval refer to these people by their role or title but do not list their name(s).

As reported on Page 11-12 of the manuscript, this study received primary ethics approval from the University of British Columbia’s Behavioral Research Ethics Board. This was an international, online study that included participants from across a broad geographical region- the 21 APEC member economies of the Asia Pacific. Because all data were collected online (using online surveys and Zoom consultations) many study partners indicated that additional ethics approvals were not required for their jurisdictions. We did however obtain ethics approval from the Malaysian Ministry of Health’s Medical Research and Ethics Committee, from Canada’s Centre for Addiction and Mental Health (CAMH) and from the Insitutional Review Board of the Institute of Population, Health and Development in Vietnam.

If there were any deviations from the study protocol after approval was obtained please provide details of these changes in the Methods section of your manuscript.
Did this study involve local collaborators that are residents of the country where the research was conducted or members of the community studied? If you do not have any authors from said communities, please provide an explanation for this below.

This study included investigators representing eight countries across the Asia Pacific region (Australia, Canada, China, Chile, Malaysia, South Korea, Vietnam, the United States). All co-investigators are included in as authors on this manuscript. Because the study consisted of online data collection targeting people living in all 21 of the APEC member economies, we do not have study team representatives/ co-authors from all countries. However this study was intended to provide a broad overview of the situation of digital mental health equity across the region as a whole and does not provide in-depth analysis of the context of individual countries or communities. We believe the diverse study team, which includes researchers at all career stages, clinicians, and policy makers from both high and low-and-middle income countries reflects our commitment to cross-regional representation and integrated knowledge translation.

Reported on page number: 9

The original intention of this study was to include the perspectives of non-English speaking populations by holding country-specific consultations in Chile, Malaysia and Vietnam. As the pandemic situation evolved and demands on Ministry of Health staff became overwhelming, it became impossible for our in-country partners in Chile and Malaysia to conduct this work. We did, however conduct in-country consultations in Vietnam in Vietnamese. These data are not included in this analysis and will be published elsewhere.

Everyone listed as an author should meet PLOS’ criteria for authorship and all individuals who meet these criteria should be included in the author byline, rather than the acknowledgements. For further information please see the journal’s Authorship Policy.

**Human subjects research (e.g. health research, medical research, cross-cultural psychology)**

Did you obtain written informed consent from a representative of the local community or region before the research took place? How did you establish who speaks for the community? Details of written informed consent obtained from study participants should be reported separately in the Methods section of your manuscript.

How did members of the local community provide input on the aims of the research investigation, its methodology, and its anticipated outcome(s)?

The study team was made up of co-investigators from eight countries and included researchers, policy makers and clinicians. All study team members contributed to study focus and design from the proposal phase through to the dissemination of results.

We obtained written informed consent from all study participants via online consent forms as described in the methods section. For the survey components of this study we used convenience sampling and asked participants to identify their country of residence in addition their experience/ affiliation(s) including whether they had lived or living experience of a mental health condition, were a clinician or other health or social service provider, and whether they were a policy maker. For the online consultations, we recruited participants using a combination of referrals from study team members, online searches of relevant organizations, and snowball sampling. While we cannot be certain that study participants were representative of their local communities or of others with the same experiences/ affiliations, we are confident that we captured a broad range of perspectives from across the region, which was our intention.

When engaging with the local community, how did you ensure that the informed consent documents and other materials could be understood by local stakeholders?

The informed consent documents and study recruitment materials were written in plain language. Due to resource limitations and the contraints of data collection during the pandemic only people who were able to participate in English were included in the study. This is described as a study limitation on Page 43.

Will the findings of the research be made available in an understandable format to stakeholders in the community where the study was conducted (e.g. via a presentation, summary report, copies of publications, etc.)? Please provide details of how this will be achieved.

In addition to the submission of this manuscript which, if successfully published, will be circulated to policy, clinical and lived experience networks throughout the region, we have presented preliminary study results to diverse communities across the region, including via an online forum hosted by the APEC Digital Hub for Mental Health on Mental Health Equity in the Asia Pacific. Preliminary results have also been presented to policy partners via the APEC Digital Hub network. Following peer review we will disseminate final results and recommendations via multiple channels, including in animated learning modules that will be co-created with a lived experience advisory council with members from across the region.

**Non-human subjects research using specimens/ animals collected as part of the study, or those housed in archival collections. Examples include archaeology, paleontology, botany and zoology.**

Did the permission you obtained from a local authority to perform the study include an agreement on access to outputs and benefit sharing? This may include procedures to enable fair distribution of the benefits and resources arising from the research performed. Please include any details of Prior Informed Consent and Benefit Sharing Agreements obtained. These may be required by field-specific regulations, for example the Convention on Biological Diversity (CBD) and the associated Nagoya Protocol.

Not applicable

If the material used in your study was imported, please A) provide the year it was imported and B) indicate whether permits were obtained to import/export the materials used, C) provide details of any permits obtained. If this information is not available, please indicate this.

Not applicable

If you used archival specimens, please state how the material used in your study was acquired by the institute it is held in and provide details of any permits obtained for the original excavations/ sample collection. If this information is not available, please indicate this.

Not applicable

How was the potential cultural significance of the materials collected in your study to local communities considered in your research design? Were Indigenous peoples and/or local researchers and institutions involved with archaeological excavations / collection of specimens? If so, please provide a description of their involvement.

Not applicable

If your manuscript includes photographs of human remains please indicate whether authors obtained permission from descendants or affiliated cultural communities to do so.

Not applicable
