# Supplementary material for: Advancing equitable access to digital mental health in the Asia-Pacific region in the context of the COVID-19 pandemic and beyond: A modified Delphi consensus study
Source: PLOS Glob Public Health. 2024 Jun 10;4(6):e0002661. doi: 10.1371/journal.pgph.0002661 (PMC11164385; doi:10.1371/journal.pgph.0002661)
Supplement: Attachment — Submitted filename: Response to Reviewers.docx [file pgph.0002661.s002.docx]

April 4, 2024

Dr. Catherine Kyobutungi and Prof. Madhukar Pai

Editors-in-Chief

PLoS Global Public Health

Dear Editors,

Thank you for the opportunity to review and resubmit the manuscript entitled “Advancing equitable access to digital mental health in the Asia-Pacific region in the context of the COVID-19 pandemic and beyond: A modified Delphi consensus study” for consideration by *PLoS Global Public Health,* and for providing some flexibility in the deadline.

We have responded to the comments from the editor and reviewer below (responses are below the original comment in **bold**). We have also made corresponding changes in the manuscript in track changes.

**Comments from the Editor:**

1. Please provide additional details regarding participant consent. In the ethics statement in the Methods and online submission information, please ensure that you have specified what type of consent you obtained (for instance, written or verbal, and if verbal, how it was documented and witnessed). If your study included minors, state whether you obtained consent from parents or guardians. If the need for consent was waived by the ethics committee, please include this information.

**We have updated the ethics statement in the manuscript (page 12) and in the online submission system to state:**

**“Both surveys included an online consent form from which written consent was obtained by requiring participants to select “yes to continue the survey” in order to access the survey. Participants selecting “no” were immediately directed to a page thanking them for their time and were unable to access survey questions.”**

2. Please include a complete copy of PLOS’ questionnaire on inclusivity in global research in your revised manuscript. Our policy for research in this area aims to improve transparency in the reporting of research performed outside of researchers’ own country or community. The policy applies to researchers who have travelled to a different country to conduct research, research with Indigenous populations or their lands, and research on cultural artefacts. The questionnaire can also be requested at the journal’s discretion for any other submissions, even if these conditions are not met.  Please find more information on the policy and a link to download a blank copy of the questionnaire here: <https://journals.plos.org/globalpublichealth/s/best-practices-in-research-reporting>. Please upload a completed version of your questionnaire as Supporting Information when you resubmit your manuscript.

**We have completed the questionnaire and have uploaded it as Supporting Information with the resubmission. We have also included the recommended text on pages 12-13 as follows:**

***“Inclusivity in global research***

***Additional information regarding the ethical, cultural, and scientific considerations specific to inclusivity in global research is included in the Supporting Information.”***

**We have also expanded on details including the ethics approval process, as follows, on pages 11-12:**

***Primary ethics approval for this study was obtained from the University of British Columbia’s (UBC) Behavioural Research Ethics Board [H20-01993]. Many of the study co-investigators indicated that ethics approval from UBC as the lead institution was sufficient given the study involved online data collection from an international sample. However, ethics approval was required and obtained from some partners, including the Malaysia Ministry of Health’s Medical Research and Ethics Committee [NMRR-20-2123-56607], the Centre for Addiction and Mental Health at the University of Toronto [109/2020] and the Vietnamese Institute of Population, Health and Development’s Institutional Review Board [2020/PHAD/DELPI-01].***

**We have also uploaded the additional ethics approval documents in the online submission.**

**We have provided the following additional detail about deviation from the original protocol, which occurred due to the rapidly evolving COVID-19 situation, as follows on Page 9:**

***“The original intention of this study was to include the perspectives of non-English speaking populations by holding country-specific consultations in Chile, Malaysia and Vietnam. As the pandemic situation evolved and demands on Ministry of Health staff became overwhelming, it was impossible for our in-country partners in Chile and Malaysia to conduct this work. We did, however conduct in-country consultations in Vietnam in Vietnamese. These data are not included in this analysis and will be published elsewhere.”***

3. Please send a completed 'Competing Interests' statement, including any COIs declared by your co-authors. If you have no competing interests to declare, please state "The authors have declared that no competing interests exist". Otherwise please declare all competing interests beginning with the statement "I have read the journal's policy and the authors of this manuscript have the following competing interests:"

**We have included the competing interests statement in the online submission system. We have updated it to include the required language as detailed above.**

4. Please amend your detailed Financial Disclosure statement. This is published with the article. It must therefore be completed in full sentences and contain the exact wording you wish to be published.

a. State the initials, alongside each funding source, of each author to receive each grant.
b. State what role the funders took in the study. If the funders had no role in your study, please state: “The funders had no role in study design, data collection and analysis, decision to publish, or preparation of the manuscript.”
c. If any authors received a salary from any of your funders, please state which authors and which funders.

If you did not receive any funding for this study, please simply state: “The authors received no specific funding for this work.

**We have updated the Financial Disclosure statement in the online submission system as follows:**

**This study was funded by a COVID-19 Knowledge Synthesis Grant from the Canadian Institutes of Health Research (FRN: 171735). RWL, JKM and EEM were co-Principal Investigators for this grant. MI, MW, CN, AG, JON, VCN, HM, AR, AP, JC, TYH, NI and SH were co-investigators for the grant. PAC and AK received salary support from this grant.**

**The APEC Digital Hub for Mental Health has received core funding from the Public Health Agency of Canada's International Health Grants Program.**

**The funders had no role in study design, data collection and analysis, decision to publish, or preparation of the manuscript.**

5. We do not publish any copyright or trademark symbols that usually accompany proprietary names, eg (R), (C), or TM  (e.g. next to drug or reagent names). Please remove all instances of trademark/copyright symbols throughout the text, including © on page 44.

**We have updated all references to remove the copyright symbol beginning on Page 44.**

6. In the online submission form, you indicated that "Data are available upon request from the corresponding author". All PLOS journals now require all data underlying the findings described in their manuscript to be freely available to other researchers, either 1. In a public repository, 2. Within the manuscript itself, or 3. Uploaded as supplementary information.
This policy applies to all data except where public deposition would breach compliance with the protocol approved by your research ethics board. If your data cannot be made publicly available for ethical or legal reasons (e.g., public availability would compromise patient privacy), please explain your reasons by return email and your exemption request will be escalated to the editor for approval. Your exemption request will be handled independently and will not hold up the peer review process, but will need to be resolved should your manuscript be accepted for publication. One of the Editorial team will then be in touch if there are any issues.

**The survey data are now publicly available on Open Science Framework (DOI 10.17605/OSF.IO/KWH52). To protect participant confidentiality, including due to concerns about the inclusion of identifiers and sensitive information in a relatively small dataset, we have not made the transcripts from the qualitative consultations publicly available.**

**Reviewer #1:**

This paper presents a modified Delphi study aimed at assessing needs and identifying priorities related to equitable digital mental health (DMH) access among marginalised populations in the Asia Pacific region during the first year of the COVID-19 pandemic. The paper is well written and structured, offering valuable insights into improving access to DMH. I commend the authors for their comprehensive approach and thoughtful analysis. However, I suggest the following for further clarification and enhancement of the manuscript:

**We wish to thank the reviewer for their positive comments and their helpful suggestions. We are including our responses below in bold and have made corresponding revisions in the manuscript, as detailed below.**

1. Rationale for Using Delphi:
The authors stated, 'Our intention was not to reach full consensus but rather to generate a comprehensive picture of the landscape, needs, and priorities related to DMH access among at-risk groups across the region in the context of an unprecedented global health emergency.' However, given that reaching consensus is not the aim of the study, it would be beneficial to discuss whether the Delphi technique remains the most suitable method or if calling it a mixed-methods study would be more appropriate.

**Thank you for your question. We have expanded the detail and justification in the Methods section (on pages 7-8) to further clarify our methodological approach and the rationale for describing the study as using a modified Delphi consensus process as follow:**

***We used a modified Delphi consensus methodology, which is effective for undertaking priority setting activities that involve diverse and geographically widespread stakeholders and when relying on online data collection methods (37-39). We followed the Delphi process of using previous rounds (both surveys and consultations) to inform subsequent survey and consultation questions in order to increasingly refine the process of priority setting, with the first survey round informed by a rapid review of the literature (1). We made several modifications to the traditional Delphi technique. In addition to reaching consensus on a topic, Delphi processes can be used for “exploration of a field beyond existing knowledge” (39). Our intention, therefore, was not to reach full consensus but rather to generate a comprehensive picture of the landscape, needs and priorities related to DMH access among at-risk groups across the region in the context of an unprecedented global health emergency in order to generate recommendations to improve DMH equity. We included three broad expert groups in this process: 1) policy makers, 2) clinicians, care, and service providers; and 3) people with lived or living experience (PWLE) of mental health conditions and their families or caregivers. An additional modification to the traditional Delphi method was to include different experts within these groups throughout the process in an effort to ensure that we captured a diversity of perspectives across a broad geographic area and from participants with varying experiences. Finally, to provide more in depth insight into the research topic, we included online consultations with expert panels in between survey rounds, drawing on the results of these consultations to inform the ranking process in the final survey, as described below.***

2. DMH Accessibility Across APEC Countries:
Could the author provide additional information regarding the accessibility of DMH services in each APEC country? Considering the significant contextual variations across countries in terms of technology ownership and DMH development, an overview of the general accessibility status in each country would enhance the contextual understanding of the study.

**Thank you for this suggestion. The APEC region is made up of 21 member economies, with varying availability of data on the accessibility of DMH in each economy. While it is not possible (and would add to the already substantial length of this manuscript) to provide data for each economy, we have introduced text in the Introduction that was previously included only in the Discussion in order to better frame the study objectives and improve contextual understanding. We have included the following text (page 6):**

***Despite the opportunities presented by the pandemic to advance evidence based DMH services, the rapid shift to DMH application raised concerns about equitable access to these technologies by many populations in the Asia-Pacific region. Although comprehensive data on DMH access across the APEC region is unavailable, evidence suggests that while digital technology use in the region is widespread, access to digital technologies in general is not equal among many sub-populations. For example, the Canadian Radio-television and Telecommunications Commission (CRTC) reports that while broadband internet is accessible to 91.4% of the general Canadian population, only 62% of Canadians in rural communities and 43.3% of First Nations have adequate broadband access (30). A study from China also found significantly lower Internet access among people in rural areas compared with people living in urban centers (31). In Indonesia, 80% of people who are not able to access the Internet live in rural areas (32). People who experience marginalization are also at risk of facing barriers to DMH access (1,33,34).***

3. Recruitment Process:
The authors recruited a large sample size, which is impressive. However, the procedures to ensure the inclusion of the 'right individuals' (i.e., those with expertise regarding the topic, such as policymakers, service users, and those with lived experiences) were not clear. I suggest providing further information regarding the eligibility criteria for study participation and the screening process.

**Thank you for your comment. We used a convenience sampling approach to recruit a broad sample of survey respondents from across the region. While convenience sampling does limit generalizability, we believe that the large sample and use of mixed methods helps to strengthen the findings. We have included more detail in the Methods section on pages 9-10 and in the Limitations section on page 42 as follows:**

**Methods**

**Study recruitment**

**We used convenience sampling to recruit for both surveys. The initial rapid survey was shared within the Digital Hub’s network via email and an online newsletter. An invitation to participate in the second survey was also disseminated among the Digital Hub network via our newsletter, as well as among the investigators’ networks by email and widely using social media. Inclusion criteria were broad to allow for diverse participation and required that participants be aged 19 years or older, be able to provide informed consent and reside in one of the 21 APEC member economies. Participants were asked to select their affiliation (see Table 2) and were able to select more than one option as appropriate.**

**Limitations**

**We used a non-probability convenience sampling approach for survey recruitment, which limits generalizability of the results. We were, however, able to recruit a substantial sample that included responses from across the APEC region and by all stakeholder groups. The mixed methods approach, which included consultations with the three broad stakeholder groups of interest, further strengthen the results by provided in-depth perspectives related to the issue of digital mental health equity across the APEC region.**

Please specify the software used for data analysis to ensure transparency and replicability.

**Statistical analysis was done using Stata 18 for Windows and qualitative analysis was completed using NVivo 12. We have added this information to the Methods section on pages 10 and 11 of the manuscript.**

5. Statistical Analysis:
I suggest the authors expand the statistical analysis to include between-group differences rather than solely comparing numbers. For example, ‘A majority indicated that they have noticed an increase, with no variation between LMIC (86.2%) and HIC (86.0%) respondents.’ (page 13, lines 327-328); ‘A majority (79.9%) responded that they had, with more people who had accessed DMH (91.2%) residing in LMICs compared with those in HICs (78.6%).’ (page 14, lines 333-334).

**Thank you for your feedback. Initially, our analysis was descriptive, and we aimed to highlight the proportion of responses from LMICs and HICs. We appreciate your suggestion to expand this analysis to include between-group differences. In response to your comment, we have conducted additional statistical tests to evaluate the significance of the differences between LMIC and HIC respondents. Specifically, we employed the Chi-square Test of Independence to examine whether the observed differences in responses were statistically significant or could have occurred by chance. The results of these tests are integrated into Table 4 (page 16), 5 (page 17), 6 (page 20), 7 (page 23), 8 (page 28) and 9 (page 32), with a description of the findings related to statistical significance integrated into the text corresponding to each table. We have also added the following text to the Methods section on page 10:**

***We conducted Chi-square Tests of Independence using STATA 18 for Windows (42) to examine whether the observed differences in responses between LMIC and HIC respondents were statistically significant.***

6. Survey 2 and Handling of Data:
Please explain the process of identifying and handling bot and spam survey responses in Survey 2, especially given the substantial number of such responses.

**Thank you for your question. We have added the following text on Page 13:**

**We cleaned the data for bots and spam responses. We initially used the built in spam and survey preview filters included in the Qualtrics survey software, which identifies likely bot, duplicate and spam responses. We also excluded responses taking less than 150 seconds (three minutes), which we deemed insufficient to properly engage with the survey.**

7. Reporting of Missing Data:
Are there missing data in the two surveys? If so, could the authors explain how missing data of the two surveys were handled?

**Thank you for your question. We have added the following text on pages 10-11 of the paper:**

***We adopted a consistent approach to managing missing data across both surveys. During our explanatory analysis we found less than 5% missing data. This, coupled with other analyses focusing on the nature and distribution of the missing data led us hold the assumption that the data were Missing Completely at Random (MCAR) (43). This assumption and the small proportion of missing data led us to use complete case analysis; for any category where responses were missing, we excluded these non-responses entirely from the analysis and did not impute missing values or assign them a default value. This decision was informed by our intention to maintain the integrity and accuracy of the data collected. This approach is supported by previous studies that showed that missing data is inevitable in mail surveys (44), complete case analysis is a common practice in epidemiological studies (45,46), and if the missing data is MCAR then complete-case analysis will result in unbiased estimate (47-49). This study employs a descriptive approach and as such, no inferential statistical method is employed or inference/generalization is claimed at the population level.***

In summary, while the study is commendable in its comprehensive approach to understanding DMH access in the Asia Pacific region during the pandemic, addressing these suggestions would enhance the clarity, transparency, and reliability of the research.

We thank the editors and reviewers for considering our resubmission.

Best wishes,

Dr. Jill Murphy

Research Associate

Department of Psychiatry, Faculty of Medicine

University of British Columbia
